# Engineering a cyanobacterium as the catalyst for the photosynthetic conversion of CO_2_ to 1,2-propanediol

**DOI:** 10.1186/1475-2859-12-4

**Published:** 2013-01-22

**Authors:** Han Li, James C Liao

**Affiliations:** 1 Department of Chemical and Biomolecular Engineering, University of California, Los Angeles, CA, 90095, USA; 2 The Molecular Biology Institute, University of California, Los Angeles, CA, 90095, USA; 3 Department of Chemistry & Biochemistry, University of California, Los Angeles, CA, 90095, USA; 4 Institute of Genomics and Proteomics, University of California, Los Angeles, CA, 90095, USA

**Keywords:** Cyanobacteria, 1,2-propanediol, Photosynthesis, CO_2_ fixation, Metabolic engineering, Synthetic biology

## Abstract

**Background:**

The modern society primarily relies on petroleum and natural gas for the production of fuels and chemicals. One of the major commodity chemicals 1,2-propanediol (1,2-PDO), which has an annual production of more than 0.5 million tons in the United States, is currently produced by chemical processes from petroleum derived propylene oxide, which is energy intensive and not sustainable. In this study, we sought to achieve photosynthetic production of 1,2-PDO from CO_2_ using a genetically engineered cyanobacterium *Synechococcus elongatus* PCC 7942. Compared to the previously reported biological 1,2-PDO production processes which used sugar or glycerol as the substrates, direct chemical production from CO_2_ in photosynthetic organisms recycles the atmospheric CO_2_ and will not compete with food crops for arable land.

**Results:**

In this study, we reported photosynthetic production of 1,2-PDO from CO_2_ using a genetically engineered cyanobacterium *Synechococcus elongatus* PCC 7942. Introduction of the genes encoding methylglyoxal synthase (*mgsA*), glycerol dehydrogenase (*gldA*), and aldehyde reductase (*yqhD*) resulted in the production of ~22mg/L 1,2-PDO from CO_2_. However, a comparable amount of the pathway intermediate acetol was also produced, especially during the stationary phase. The production of 1,2-PDO requires a robust input of reducing equivalents from cellular metabolism. To take advantage of cyanobacteria’s NADPH pool, the synthetic pathway of 1,2-PDO was engineered to be NADPH-dependent by exploiting the NADPH-specific secondary alcohol dehydrogenases which have not been reported for 1,2-PDO production previously. This optimization strategy resulted in the production of ~150mg/L 1,2-PDO and minimized the accumulation of the incomplete reduction product, acetol.

**Conclusion:**

This work demonstrated that cyanobacteria can be engineered as a catalyst for the photosynthetic conversion of CO_2_ to 1,2-PDO. This work also characterized two NADPH-dependent sADHs for their catalytic capacity in 1,2-PDO formation, and suggested that they may be useful tools for renewable production of reduced chemicals in photosynthetic organisms.

## Background

Many natural metabolites containing bi-functional groups such as succinate, lactate, and 3-hydroxybutaoate can be used as monomers to make polymers, and have long been produced biologically by fermentation processes. However, production of diols from renewable source represents unique challenges partially because they are not typical fermentation products and that they are more reduced compared to the average carbon redox state in biological systems [1]. Over the past decade, progress in synthetic biology and metabolic engineering have enabled substantial achievements in diol production [1-4], mostly from sugars, glycerol, or biomass feedstocks. Direct production of chemicals from CO_2_ in photosynthetic organisms [5-7] and lithoautotrophic organisms [8-10] have been proposed to be advantageous in particular situations. This work aims to produce 1,2-propanediol (1,2-PDO) directly from CO_2_ by an engineered cyanobacterium, *Synechococcus elongatus* PCC 7942.

**Figure 1:**
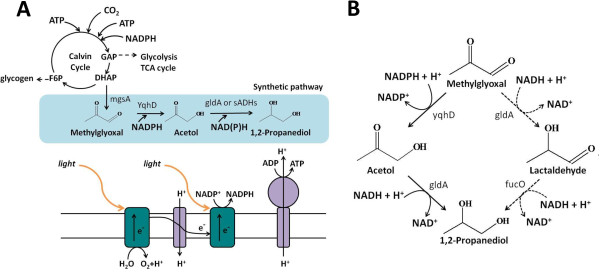
**The pathway for 1,2-Propanediol (1,2-PDO) production from CO**_**2 **_**in *Synechococcus elongatus *PCC 7942. A**) Light reaction of photosynthesis generates ATP and reducing equivalents NADPH, which power the CO_2_ fixation through Calvin cycle and the synthetic 1,2-PDO production pathway. *gldA*, *yqhD* and *mgsA* are from E.coli. Secondary alcohol dehydrogenases (*sADHs*) are from *C. beijerinckii* and *T. brockii*. DHAP, dihydroxyacetone phosphate. GAP, glyceraldehyde-3-phosphate. F6P, fructose-6-phophate. **B**) Two possible pathways for 1,2-Propanediol synthesis from methylglyoxal.

The racemic 1,2-PDO can find applications in antifreeze and heat transfer fluids, plasticizers and thermoset plastics, and cosmetics [11]. 1,2-PDO is naturally produced by anaerobic microorganisms such as *Thermoanaerobacterium thermosaccharolyticum*[12]. It has also been produced by engineered *E.coli*[3,11,13], *Corynebacterium glutamicum*[14], *Saccharomyces cerevisiae*[15,16], and *Pichia Pastoris*[17] from glycerol or sugar. Consistent with the fermentative nature of the pathway in its native host, 1,2-PDO production in heterologous hosts only achieved relatively high titer (4.5 ~ 6.5g/L) in anaerobic fermentation. However, cyanobacterium *S. elongatus* produces O_2_ in the light reaction of photosynthesis (Figure 1A) and does not perform fermentation in light. Moreover, CO_2_ is a more oxidized substrate than sugar or glycerol and therefore requires more energy and reducing equivalents to be converted to the product. Here we report introduction of the heterologous 1,2-PDO production pathway into *S. elongatus* and subsequent tailoring of the pathway to fit in the metabolism of this photosynthetic CO_2_-fixing host.

## Results and discussion

### Designing of the 1,2-PDO production pathway

In light conditions, cyanobacteria fix CO_2_ via the Calvin-Benson-Bassham (CBB) cycle which is powered by ATP and NADPH generated by the photosystems (Figure 1A). Two CBB cycle intermediates, fructose-6-phosphate (F6P) and glyceraldehydes-3-phosphate (GAP), serve as the branch points of carbon leaving the CBB cycle to the central metabolism for glycogen synthesis and glycolysis, respectively. While glycogen synthesis is the major carbon and energy storage pathway, glycolysis and TCA cycle produce building blocks for cell growth. The synthesis of 1,2-PDO, on the other hand, starts from another CBB cycle intermediate, dihydroxyacetonephosphate (DHAP). The introduction of one extra branch point can potentially increase the flux of output carbon from the CBB cycle, which has been suggested to be beneficial for increasing photosynthesis efficiency in higher plants [18,19], but may also disrupt the normal flux distribution in the cell.

To synthesize 1,2-PDO, DHAP is first converted to methylglyoxal (Figure 1A) by methylglyoxal synthase (encoded by *mgsA* in *E. coli*). Methyglyoxal is very toxic to the cells [11] and needs to be efficiently utilized by downstream enzymes. Two different metabolic routes have been shown to synthesize 1,2-PDO from methyglyoxal (Figure 1B) [11]. The first involves reduction of methyglyoxal by the glycerol dehydrogenase (encoded by *gldA* in *E. coli*) to lactaldehyde, which is further reduced by the 1,2-propanediol reductase (encoded by *fucO* in *E.coli*) to yield the final product. The second route includes an alcohol dehydrogenase (such as the broad-substrate range aldehyde reductase encoded by *yqhD* in *E. coli*) to produce acetol as the intermediate, which is then converted to 1,2-PDO by gldA. The latter route was chosen to introduce into *S. elongatus* because *yqhD* gene has been previously overexpressed in this organism for biofuel production and showed relatively good performance, possibly due to its NADPH-specific cofactor preference.

### Introduction of the 1,2-PDO biosynthesis genes

**Figure 2:**
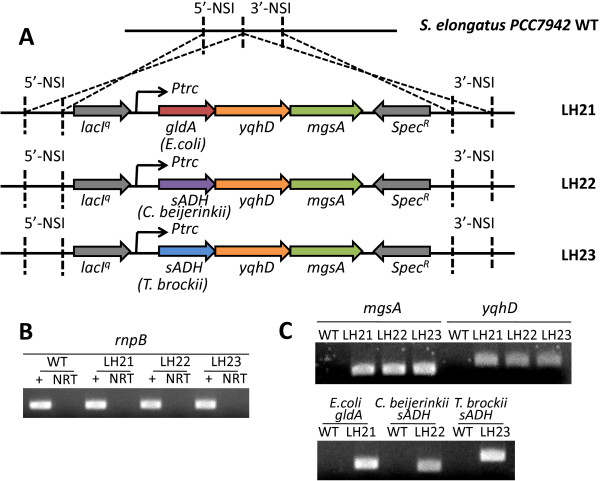
**Construction of the 1,2-propanediol pathways. **(**A**) Illustration of artifical operons inserted into the *Synechococcus elongatus* PCC 7942 Neutral Site I (NSI) to build strains LH21, LH22, and LH23. *gldA*, *yqhD* and *mgsA* are from E.coli. Secondary alcohol dehydrogenases (*sADHs*) are from *C. beijerinckii* and *T. brockii*. (**B**) Test of the total RNA quality and the RT-PCR system using housekeeping gene *rnpB*. (**C**) Verification of the heterologous gene expression in engineered cyanobacteria strains by RT-PCR.

As described above, the genes *mgsA*, *yqhD*, and *gldA* from *E. coli* are needed to construct the 1,2-PDO biosynthesis pathway in *S. elongatus* from the CBB cycle intermediate DHAP (Figure 1A, B). These genes were cloned into an artificial operon driven by the P_trc_ promoter under the control of lacO (Figure 2A). The operon was inserted into the *S. elongatus* chromosome by homologous recombination at the Neutral Site I (NSI). A *lacI* gene and a spectinomycin resistant gene were also inserted together with the operon to achieve inducible gene expression (Additional file 1: Figure S2) and facilitate antibiotics selection, respectively. The resulting strain was named LH21.

**Figure 3:**
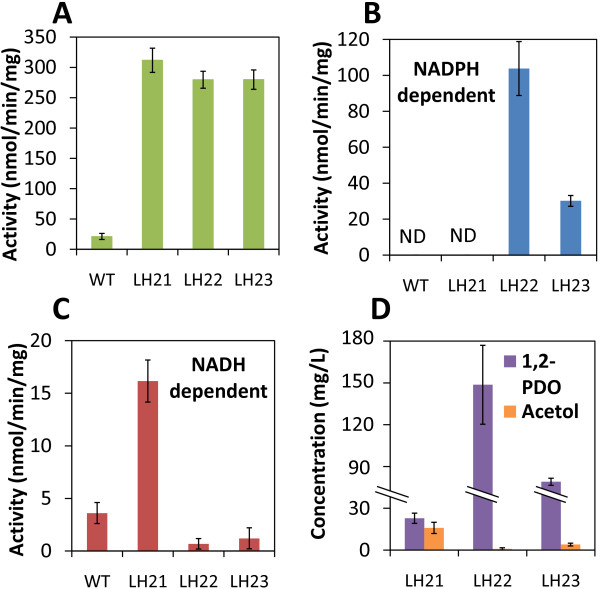
**Optimization of the 1,2-Propanediol production by exploiting different acetol-reducing enzymes. **LH22 and LH23 were constructed which harbored *C. beijerinckii* and *T. brockii*. sADH. **A**) methylglyoxal synthase activities were measured using cell lysate of LH21, LH22, and LH23, showing that *mgsA* was functionally overexpressed in all three strains. To confirm the functional expression of *yqhD*, NADPH-dependent methylglyoxal reduction activities were also measured (See Additional file 1: Figure S1 and text in supporting material), although assay method with higher specificity needs to be developed. (**B**), (**C**) NADPH- and NADH-dependent acetol reduction activities measured using cell lysates of the wildtype *Synechococcus elongatus* PCC 7942 and engineered strains that overexpress *gldA* from *E.coli* (LH21), *adh* from *C. beijerinckiiadh* (LH22), and *adh* from *T. brockiadh* (LH23). (D) Acetol and 1,2-Propanediol accumulated by different strains after 10 days of production.

To check if all the genes were successfully introduced and transcribed, reverse transcription polymerase chain reaction (RT-PCR) was performed. After induction with Isopropyl β-D-1-thiogalactopyranoside (IPTG), total RNA was extracted from LH21 and wildtype *S. elongatus PCC 7942*. RT-PCR of the house keeping gene *rnpB*, whose transcription product is the RNA component of RNase P, was performed to verify the RT-PCR system. Using the *rnpB* specific primers, PCR products were obtained using cDNA synthesized from both wildtype and LH21 total RNA (Figure 2B). On the other hand, the no-reverse-transcriptase (NRT) controls did not yield any products, suggesting that the genomic DNA contamination during the total RNA extraction was minimal and that the positive signals of RT-PCR are representative for the transcription of the target genes. Using the verified system, *mgsA*, *yqhD*, and *gldA* genes from *E. coli* were tested and showed to have expression in LH21 under inductive condition (Figure 2C). Activity assays using cell lysate further suggested that these heterologous enzymes were functional in cyanobacterial cells (Figure 3A, B, Additional file 1: Figure S1).

### Production of 1,2-PDO

**Figure 4:**
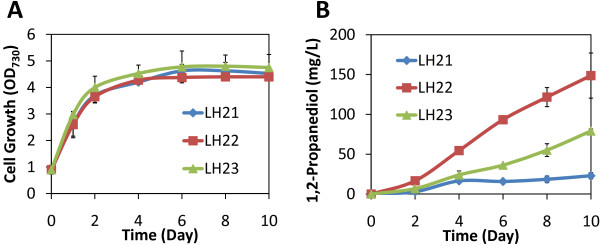
Cell growth and 1,2-Propanediol production by engineered Cyanobacteria strains.

1,2-PDO production by LH21 was performed under high light condition (100 μE/s/m^2^) with 50mM bicarbonate supplementation in the medium. After induction, LH21 produced around 16mg/L 1,2-PDO in 4 days, with the highest production rate of 7mg/L/day. However, although no defect in cell growth was seen, the production rate decreased rapidly and the total titer was only around 22mg/L after 10 days (Figure 4A, B). The drastically decreased and eventually ceased production by LH21 led to one hypothesis: key substrate(s) might become limited at certain stage of cell growth which decreased the flux of the synthetic 1,2-PDO production pathway. Three substrates are needed to produce 1,2-PDO in LH21: NADPH, NADH, and DHAP (Figure 1A, B). Among these substrates, NADPH and DHAP are made through photosynthesis light and dark reactions (Figure 1A), respectively, and could be continuously generated under light condition. However, NADH may mainly be generated by the NADH-dependent glyceraldehyde-3-phosphate dehydrogenase in the glycolysis [20]. And the reaction catalyzed by the putative NADH-dependent malate dehydrogenase in TCA cycle may also contribute to the cellular NADH level. It has been suggested that the main function of glycolysis and TCA cycle in cyanobacteria is to generate essential metabolites for biomass synthesis under light conditions, rather than to produce reducing equivalents and energy [21]. As such, when the growth rate of LH21 cells slowed down in the stationary phase, the activities of these NADH generating pathways may also decrease.

If NADH is really the limiting factor in our production scenario, the partially reduced intermediate acetol may accumulate. In fact, at the end of the production, around 16mg/L acetol was accumulated, which was comparable to the level of 1,2-PDO (~22mg/L) (Figure 3D). In addition, acetol was only detected after 4 days and kept accumulating during the late stage of production (data not shown). These results are consistent with the above-mentioned hypothesis and suggest that the NADH-dependent reduction of acetol catalyzed by gldA might be the limiting step in the 1,2-PDO production pathway.

### Improving 1,2-PDO production using NADPH-dependent secondary alcohol dehydrogenases

To overcome the bottleneck of the 1,2-PDO production in LH21, one possible strategy is to overexpress the soluble transdehydrogenase (STH) which produces NADH at the expense of NADPH. However, genes encoding this enzyme have not been found in *S. elongatus* genome. Heterologous overexpression of the *Pseudomonas aeruginosasth* gene in cyanobacteria has been shown to be instable and caused growth defect [22].

Alternatively, it could be beneficial to find the NADPH-dependent counterpart of gldA. To convert acetol to 1,2-PDO, a hydroxyl group on the secondary carbon has to be made, which can be catalyzed by the secondary alcohol dehydrogenase (sADH) family of enzymes. Several sADHs have been characterized previously that are NADPH-dependent [4], including the sADH encoded by the *adh* gene in *Thermoanaero bacterbrockii*[23] and *Clostridium beijerinckii*[24]. To test their catalytic activity for the substrate acetol, these two sADHs as well as the *E. coli* gldA were purified and their kinetics parameters were measured (Table 1). The kinetics studies showed that the *C. beijerinckii* sADH has the highest *K*_*cat*_ among all three enzymes tested. However, the large *K*_*m*_ value of *C. beijerinckii* sADH suggested that the enzyme has relatively low affinity to the substrate acetol. On the other hand, the *T. bacterbrockii* sADH has the highest acetol affinity and a two-fold higher *K*_*cat*_ than that of the gldA enzyme, which is the most commonly used enzyme for this reaction step in previous studies. In summary, these two sADH have distinct kinetic features and both showed activity towards the substrate acetol, which suggested that they may be used in cyanobacterial cells for 1,2-propanediol production *in vivo*.

**Table 1:** Kinetics parameters of gldA and secondary alcohol dehydrogenases (sADH) for acetol

**Enzyme**	**Cofactor**	***K***_***m ***_**(mM)**	***K***_***cat ***_**(S**^**-1**^**)**	***K***_***cat***_***/K***_***m ***_**(mM**^**-1**^**S**^**-1**^**)**
*E.coli* gldA	NADH	1.64	0.59	0.36
*C.beijerinckii* sADH	NADPH	7.98	4.95	0.62
*T.brockii* sADH	NADPH	0.23	1.26	5.48

*C. beijerinckii* and *T. brockii* adh were cloned and introduced into the cyanobacterial genome to replace *gldA*. The resulting strains are named LH22 and LH23, respectively (Figure 2A). RT-PCR was also performed to verify the expression of these genes (Figure 2B, C). Enzyme assays with crude cell extract of LH22 and LH23 further verified that both *C. beijerinckii* and *T. brockii* sADH were functionally overexpressed and showed higher activities of NAD(P)H-dependent acetol reduction compared to that in LH21. Especially, the *C. beijerinckii* sADH overexpression in LH22 delivered the highest activity (Figure 3B, C).

Production using strains LH22 and LH23 yielded significantly higher 1,2-PDO titer (~150 and 80mg/L, respectively) compared to that of LH21 (Figure 4B). Notably, the high production rate was maintained through the 10 days of production. In consistent with the hypothesis mentioned in the previous section, the high level of NADPH-dependent acetol reduction activity in LH22 and LH23 also significantly reduced the accumulation of the intermediate acetol (Figure 3D).

Despite its great significance to metabolic engineers, the information on intracellular NAD(P)H level during different growth phases and growth conditions in cyanobacteria is very limited. Although it is believed that NADPH is more abundant than NADH in cyanobacteria [25], only a few studies discussed its role in biofuel/biochemical synthesis from CO_2_[6,22]. The science behind efficient conversion of CO_2_ to chemicals and fuels is still in its infancy and the NADPH driving force theory still needs to be extensively tested, which requires the accumulation of empirical evidence in more production scenario, as well as fundamental studies on NAD(P)H levels and their regulation. In our case, other factors may also contribute to the difference between the production levels of the NADH and NADPH-dependent pathways. For example, the NADPH-dependent enzymes may be better folded and more active when expressed in cyanobacteria. And different level of physiological fitness may be caused by overespression of different enzymes, although all production strains showed the same growth phenotype as the wildtype.

## Conclusion

In this work, we demonstrated the 1,2-PDO production from CO2 for the first time by the engineered cyanobacterium *S. elongatus PCC 7942*. By exploiting sADHs which have not been reported for 1,2-PDO production previously, a completely NADPH dependent pathway was built to channel the CBB cycle intermediate DHAP for 1,2-PDO production without accumulating the pathway intermediate, acetol. The best strain LH22, which harbors *mgsA* and *yqhD* both from *E. coli* and the *adh* from *C. beijerinckii*, produced ~150mg/L 1,2-PDO.

This work revealed the great potential of the vast NADPH pool in photosynthetic cyanobacteria as a robust driving force for the production of chemicals. Among the chemicals that have been produced biologically in industrial scale, a significant number of them are synthesized by NADPH consuming pathways. For example, in amino acid production, studies have shown that increasing the NADPH pool can improve the production performance [26,27]. However, in most of the heterotrophic microorganisms, NADPH is mainly generated through the pentose phosphate pathway and TCA cycle and its pool size is relatively small compared to that of the NADH. On the other hand, photosynthetic organisms maintain high intracellular NADPH level. The unique metabolic feature of photosynthetic organisms provides great opportunities for the production of chemicals through NADPH dependent pathways.

## Methods

### Chemicals and reagents

All chemicals were purchased from Sigma-Aldrich (St. Louis, MO) or Fisher Scientifics (Pittsburgh, PA). Restriction enzymes were purchased from New England BioLabs (Ipswich, MA). The Rapid DNA ligation kit was from Roche (Mannheim, Germany). KOD DNA polymerase was from EMD Chemicals (San Diego, CA). Oligonucleotides were purchased from IDT (San Diego, CA).

### Medium and culture condition

All *S. elongatus PCC 7942* strains were grown on BG-11 medium (Sigma-Aldrich) containing 50mM NaHCO_3_ in shake flasks. Plates contain 1.5% (w/v) agar. For 1,2-PDO production, 50mL culture was grown in 250mL shake flasks under 100 μE/s/m^2^ light supplied by four Lumichrome F30W-1XX 6500K 98CRI light tubes, at 30°C. Cell growth was monitored by measuring OD_730_. 1mM IPTG was added to induce the gene expression at OD_730_ of around 1. Daily, samples were taken for analysis and 50mM NaHCO_3_ was added. IPTG concentration in the culture was maintained to 1mM by adding appropriate amount of fresh IPTG to compensate the IPTG lost from sampling. Spectinomycin was added for LH21, LH22, and LH23 at a final concentration of 20mg/L. *E. coli* strains were grown in LB medium. And a spectinomycin concentration of 50mg/L was used where appropriate.

### Plasmid construction

All cloning and plasmid preparation were done using *E. coli* XL1-blue cells (Stratagene, La Jolla, CA). Detained information about plasmids and primers used in this study can be found in Table 2 and Table 3.

**Table 2:** Plasmids used in this study

**Plasmid Name**	**Description**	**Used for strain**	**Reference**
pAM2991	NSI targeting vector	-	[28]
pZE12-	Contain *C. beijerinckii adh* gene codon optimized for *E.coli*	-	[4]
alsS-alsD-CBADH			
pZE12-	Contain *T. brockii adh* gene codon optimized for *E.coli*	-	[4]
alsS-alsD-TBADH			
GYM	NSI targeting. LacI^q^; *Ptrc*::*gldA*, *yqhD*, *mgsA*; *Spec*^*R*^	LH21	This work
CYM	NSI targeting. LacI^q^; *Ptrc*:: *C.beijerinckii adh*, *yqhD*, *mgsA*; *Spec*^*R*^	LH22	This work
TYM	NSI targeting. LacI^q^; *Ptrc*::*T.brockii adh*, *yqhD*, *mgsA*; *Spec*^*R*^	LH23	This work
pZElac	*E.coli* vector whose backbone was used to build his tag protein expression plasmids used in this study.	-	[29]
pQE-9	Vector for N-terminal 6Xhis-tagged protein expression	-	Qiagen
pZElac_his	The pZElac inserted with the T5 promoter/lacO::6Xhis part from pQE-9.	-	This work
His-gldA	*E.coli* vector for his tag- *E.coli* gldA expression.	-	This work
His-CB	*E.coli* vector for his tag- *C. beijerinckii* adh expression.	-	This work
His-TB	*E.coli* vector for his tag- *T. brockii* adh expression.	-	This work

**Table 3:** Primers used in this study

**Primer Name**	**Sequence (5**^**′**^**-3**^**′**^**)**	**Used for plasmid**
gldASpeIfwd	aggatcactagtaggagaagttaccatggaccgcattattcaatcaccgg	GYM
gldA_YqhDrev	ggtgtgcagattaaagttgttcatggtaacttctcctttattcccactcttgcaggaaac	GYM
gldA_YqhDfwd	gtttcctgcaagagtgggaataaaggagaagttaccatgaacaactttaatctgcacacc	GYM
YqhD_mgsArev	aaagtgcgagtcgtcagttccatggtaacttctcctttagcgggcggcttcgtatatacg	GYM
YqhD_mgsAfwd	cgtatatacgaagccgcccgctaaaggagaagttaccatggaactgacgactcgcacttt	GYM
mgsANotI rev	ggatcggcggccgcttacttcagacggtccgcgagataacgctga	GYM
CBSADH SpeIfwd	aggatcactagtaggagaagttaccatgaaagggtttgccatgttag	CYM
CBSADH_yqhDrev	gcagattaaagttgttcatggtaacttctcctttacaggataacaaccgc	CYM
CBSADH_yqhDfwd	gcggttgttatcctgtaaaggagaagttaccatgaacaactttaatctgc	CYM
TBSADH SpeIfwd	aggatcactagtaggagaagttaccatgaaaggttttgcaatgctgtc	TYM
TBSADH_yqhDrev	gcagattaaagttgttcatggtaacttctcctctatgctaaaatcaccac	TYM
TBSADH_yqhDfwd	gtggtgattttagcatagaggagaagttaccatgaacaactttaatctgc	TYM
pQE XhoI fwd	ttcacctcgagaaatcataaaaaatttatttgc	pZElac_his
pQE Acc65I rev	gttttcggatccgtgatggtgatggtgatgcgatcctctc	pZElac_his
his ad up rev	ggatccgtgatggtgatggtgatgcgatcc	his-gldA/CB/TB
his ad down fwd	tctagaggcatcaaataaaacgaaaggctc	his-gldA/CB/TB
his ad up_gldA fwd	gcatcaccatcaccatcacggatccatggaccgcattattcaatcaccgg	his-gldA
his ad down_gldA rev	cctttcgttttatttgatgcctctagattattcccactcttgcaggaaac	his-gldA
his ad up_CB fwd	atcaccatcaccatcacggatccatgaaagggtttgccatgttaggtatc	his-CB
his ad down_CB rev	tttcgttttatttgatgcctctagattacaggataacaaccgccttaatc	his-CB
his ad up_TB fwd	cgcatcaccatcaccatcacggatccatgaaaggttttgcaatgctgtcc	his-TB
his ad down_TB rev	ctttcgttttatttgatgcctctagactatgctaaaatcaccactggtt	his-TB
rnpBfwd	aggtgttggctcggtaaac	RT-PCR rnpB
rnpB rev	cgaagacagagggcagttatc	
gldAfwd	gaaaccgtagctgcccttag	RT-PCR gldA
gldA rev	ggcatgttgtgaatggtttc	
mgsAfwd	tgacgactcgcactttacct	RT-PCR mgsA
mgsA rev	cgtgttgttccagtaacggt	
yqhDfwd	cgaacaaattcctcacgatg	RT-PCR yqhD
yqhD rev	tcatcagcgtttcataagcc	
CB fwd	ctccagtgtggtggttatcg	RT-PCR CBsADH
CB rev	ttagctgcttcaacgcaaat	
TB fwd	tatggtgctaccgacatcgt	RT-PCR TBsADH
TB rev	taattgacgtttgcgatggt	

Briefly, to construct plasmid GYM, *gldA*, *mgsA*, and *yqhD* were amplified from *E. coli* genomic DNA using primer pairs gldA SpeI fwd/gldA_YqhD rev, gldA_YqhD fwd/YqhD_mgsA rev, and YqhD_mgsA fwd/mgsA NotI rev, respectively. The PCR products were purified and linked into an artificial operon using Splicing by overhang extension (SOE) PCR using primers gldA SpeI fwd/mgsA NotI rev. The PCR product was digested with restriction enzymes SpeI and NotI and then inserted into the NSI targeting vector pAM2991. The CYM and TYM plasmids were constructed similarly. The *C. beijerinckii adh* and *T. brockii adh* genes were amplified from plasmids pZE12-alsS-alsD-CBADH and pZE12-alsS-alsD-TBADH [4], respectively, using primer pairs CBSADH SpeI fwd/CBSADH_yqhD rev and TBSADH SpeI fwd/TBSADH_yqhD rev, respectively. In order to amplify *yqhD* gene that have overlapping region with the *C. beijerinckii adh* and *T. brockii adh*, the forward primer for yqhD amplification was CBSADH_yqhD fwd and TBSADH_yqhD fwd, respectively.

To purify the 6xHis-tagged gldA and secondary alcohol dehydrogenases, plasmids his-gldA, his-CB, and his-TB were constructed. Briefly, the T5 promoter/lacO and 6xHis tag fragment of pQE-9 (Qiagen) was amplified using primers pQE XhoI fwd and pQE Acc65I rev and then digested and inserted at XhoI/Acc65I sites of the plasmid pZElac [29]. The resulted plasmid was named pZElac-his. To insert the *E. coli gldA*, *C. beijerinckii adh* and *T. brockii adh* genes in pZElac-his, the isothermal DNA assembly method [30] was used. The primers his ad up rev and his ad down fwd were used to amplify the vector backbone using pZElac-his as template. And the primer pairs his ad up_gldA fwd/his ad down_gldA rev, his ad up_CB fwd/his ad down_CB rev, and his ad up_TB fwd/his ad down_TB rev were used to amplify the corresponding genes. The gene amplification products were assembled with the backbone.

### Protein purification and enzyme kinetics study

The plasmid his-gldA, his-CB, and his-TB were transformed into BL21 cells. The transformants were cultured in 40 mL LB medium containing 100mg/L ampicillin. After the cells reached mid-log phase, 1mM IPTG was added to induce protein expression followed by incubation at 30°C overnight. The cells were collected by centrifugation and the recombinant proteins were purified using His-Spin Protein Miniprep kit (Zymo research Corporation, CA) according to the manufacturer’s instructions. The purified proteins were checked by SDS-PAGE for homogeneity and quantified by Bradford assay (Bio-Rad, Hercules, CA).

Dehydrogenase activity was measured by monitoring the absorbance decrease of NADH or NADPH at wavelength of 340 nm. To determine the kinetic parameters, the assay reaction was prepared with Tris-HCl buffer (50 mM, pH = 7.5) containing 200 μM of NADH or NADPH and various concentration of acetol ranging from 0.05 mM to 20 mM at room temperature. The *K*_*m*_ and *K*_*cat*_ values were obtained by non-linear fitting with the Michaelis–Menten equation.

### Transformation and selection

*S. elongatus PCC7942* cells transformed as described [31]. The transformed cells were spread on BG-11 plates with 20mg/ml spectinomycin and incubated in light to select for recombinants. Colonies were verified by PCR and inoculated into liquid BG-11 medium with 20 mg/ml spectinomycin for further tests.

### Enzyme assays

For sADH enzyme assays, different cyanobacterial strains were grown in 30mL BG-11 medium in 125mL shake flask under light condition and induced with 1mM IPTG at the OD_730_ of around 1. After overnight induction, cells were harvested and resuspended in 1mL Buffer A (100mM Tris-HCl, pH = 8.0). Cell lysates were prepared by bead beating followed by centrifugation at 10,000 × g for 20min at 4°C. Cell. 10μL cell lysate was used in 200μL reaction system which also contained 100mM Tris-HCl, pH = 8.0, 200μL NAD(P)H, and 20mM acetol (or no substrate for negative control). The reaction was started by adding the substrate, and the OD_340_ was monitored. The soluble protein concentrations in cell lysates were quantified using Quick Start Bradford Protein Assay (Bio-Rad, CA) according to manufacturer’s instructions. Similar method was used to determine the activity of methylglyoxal reduction.

The activity of methylglyoxal synthase was determined by the previously reported method [32]. Briefly, the reaction mixture at 30°C contained, in 0.5 ml: Tris-HCl buffer, pH7.5 (50mM), dihydroxyacetone phosphate (20mM) and cell lysate. The reaction was allowed to proceed for 10min. The methylglyoxal formed was measured colorimetrically by taking 0.1 ml samples into 0.33 ml of 2,4-dinitrophenylhydrazine reagent (0.1% 2,4-dinitrophenylhydrazine in 2M-HCI) plus 0.9ml of water. After incubation at 30°C for 15min, 1.67ml of 2.5M NaOH was added and the OD_555_ measured after a further 15min. A molar extinction coefficient of 4.48 × 10^4^ was used to convert the readings into nmol of methylglyoxal.

### RT-PCR

For RT-PCR enzyme assays, different cyanobacterial strains were grown in 30mL BG-11 medium in 125mL shake flask under light condition and induced with 1mM IPTG at the OD_730_ of around 1. After overnight induction, total RNA was extracted using RiboPure-Bacteria Kit (Life Technologies, NY). RNA was quantified using Nanodrop. After treatment with the TURBO DNA-free kit (Life Technologies, NY), cDNA was synthesized using iScript cDNA Synthesis kit (Bio-Rad, CA). PCR was performed using the specific primers listed in Table 2. The PCR product was then checked by electrophoresis on 2% agarose gel and stained with ethidium bromide.

### Quantification of 1,2-PDO and acetol

1mL samples were taken daily from production culture. After centrifugation, the supernatant was taken for 1,2-PDO and acetol analysis by Agilent 1200 highpressure liquid chromatography (HPLC) system equipped with an autosampler (Agilent Technologies),a Bio-Rad (Bio-Rad Laboratories, Hercules, CA) Aminex HPX87 column (5 mM H_2_SO_4_, 0.6 mL/min, column temperature at 35°C) and a refractive index detector (RID) module.

## Competing interests

The authors declare that they have no competing interests.

## Authors’ contribution

HL designed and performed the experiments and analyzed the data. HL and JCL prepared the manuscript. Both authors read and approved the final manuscript.

## Supplementary Material

Additional file 1 Measurement of the NADPH-dependent methylglyoxal reduction activity in production strains and inducibility of the synthetic pathways. Click here for file
