# Supplementary material for: Engineering a cyanobacterium as the catalyst for the photosynthetic conversion of CO2 to 1,2-propanediol
Source: Microb Cell Fact. 2013 Jan 22;12:4. doi: 10.1186/1475-2859-12-4 (PMC3556108; doi:10.1186/1475-2859-12-4)
Supplement: Additional file 1 — Measurement of the NADPH-dependent methylglyoxal reduction activity in production strains and inducibility of the synthetic pathways. [file 1475-2859-12-4-S1.docx]

**Supporting material**

**Measurement of the NADPH-dependent methylglyoxal reduction activity in production strains**

The *yqhD* gene has been overexpressed in the *Synechococcus elongatus* PCC 7942 and showed relatively high activity [S[1](#_ENREF_1)]. To confirm the functional overexpression in the 1,2-propandieol production strains, the activity of NADPH-dependent reduction of methylglyoxal was also measured (Figure S1). In LH21 strain, the measured activity was significantly higher than that in the wildtype. This difference in activity levels should represent the activity of the heterologous expressed yqhD in LH21, since the overexpresssed gldA in the same strain is NADH-dependent and would have little effect on the measurement due to the lack of NADH cofactor in the reaction system.

In LH22 and LH23 strains, the assay may be affected by the activity of NADPH-dependent secondary alcohol dehydrogenases, which can potentially also catalyze the NADPH-dependent primary alcohol formation because of their promiscuity [S[2](#_ENREF_2), S[3](#_ENREF_3)] and/or further reduce the product of yqhD reaction using NADPH. This may contribute to the higher apparent activities in LH22 and LH23, as these side reactions also consume NADPH and affect the absorbance at 340nm (see Method).

However, several lines of evidence may imply that *yqhD* is also functionally overexpressed in LH22 and LH23. First, since the last gene in the operon, *mgsA*, is suggested to be overexpressed with similar levels in all three strains (Figure 4A), the transcriptional level and mRNA stability of the whole synthetic operon should be similar in all three strains. Second, because the *yqhD* genes in all three strains have identical sequence and ribosomal-binding site (RBS) and are located at the same position in the synthetic operon (Figure 2A), the translational level of this gene and the folding behavior of its product might be similar in all three strains. However, to directly pinpoint the activity level of yqhD in these strains, a more specific activity assay other than the NADPH-consumption based assay needs to be developed.

Figure S1 NADPH-dependent methyglyoxal reduction activities measured using cell lysates.

**Inducibility of the synthetic pathways**

As an example, the activity of *Clostridium beijerinckii* secondary alcohol dehydrogenase (sADH) for the substrate acetol in LH22 was measured with and without IPTG induction (Figure S2). The results suggested that IPTG effectively induced the expression of this enzyme. And since identical promoter/operator/repressor setup was used in all three strains, same induction effect can be implied for other genes.

Figure S2 The *Clostridium beijerinckii* secondary alcohol dehydrogenase activity measured using cell lysate of LH22 with and without 1mM IPTG induction. The activity was measured for the substrate acetol.

Reference

S1. Atsumi S, Higashide W, Liao JC: **Direct photosynthetic recycling of carbon dioxide to isobutyraldehyde**. *Nat Biotechnol* 2009, **27**(12):1177-1180.

S2. Lamed RJ, Zeikus JG: **Novel NADP-linked alcohol--aldehyde/ketone oxidoreductase in thermophilic ethanologenic bacteria**. *Biochem J* 1981, **195**(1):183-190.

S3. Ismaiel AA, Zhu CX, Colby GD, Chen JS: **Purification and characterization of a primary-secondary alcohol dehydrogenase from two strains of Clostridium beijerinckii**. *J Bacteriol* 1993, **175**(16):5097-5105.
